# The odyssey of the TR(i)P journey to the cellular membrane

**DOI:** 10.3389/fcell.2024.1414935

**Published:** 2024-07-23

**Authors:** Bastián Rivera, Octavio Orellana-Serradell, Evrim Servili, Rodrigo Santos, Sebastián Brauchi, Oscar Cerda

**Affiliations:** ^1^ Program of Molecular and Cellular Biology, Institute of Biomedical Sciences, Faculty of Medicine, Universidad de Chile, Santiago, Chile; ^2^ Millennium Nucleus of Ion Channel-Associated Diseases (MiNICAD), Santiago, Valdivia, Chile; ^3^ Institute of Health Sciences, Universidad de O’Higgins, Rancagua, Chile; ^4^ Institute of Physiology, Faculty of Medicine, Universidad Austral de Chile, Valdivia, Chile

**Keywords:** TRP channels, trafficking, post-translational modifications, protein-protein interaction (PPI), ion channel mutations

## Abstract

Ion channels are integral membrane proteins mediating ion flow in response to changes in their environment. Among the different types of ion channels reported to date, the super-family of TRP channels stands out since its members have been linked to many pathophysiological processes. The family comprises 6 subfamilies and 28 members in mammals, which are widely distributed throughout most tissues and organs and have an important role in several aspects of cellular physiology. It has been evidenced that abnormal expression, post-translational modifications, and channel trafficking are associated with several pathologies, such as cancer, cardiovascular disease, diabetes, and brain disorders, among others. In this review, we present an updated summary of the mechanisms involved in the subcellular trafficking of TRP channels, with a special emphasis on whether different post-translational modifications and naturally occurring mutagenesis affect both expression and trafficking. Additionally, we describe how such changes have been associated with the development and progress of diverse pathologies associated with the gain or loss of functional phenotypes. The study of these processes will not only contribute to a better understanding the role of TRP channels in the different tissues but will also present novel possible therapeutic targets in diseases where their activity is dysregulated.

## Introduction

### The super-family of TRP ion channels

The TRP superfamily of ion channels is composed of 28 members that are subdivided into six subfamilies in mammals. TRP channels are characterized by their low sequence homology and family clustering based on overall structural similarities (Clapham, 2003; Clapham et al., 2003; Venkatachalam and Montell, 2007; Cabezas-Bratesco et al., 2022).

The different TRP subfamilies are TRPC (canonical), TRPV (vanilloid), TRPM (melastatin), TRPA (ankyrin), TRPP (polycystin), and TRPML (mucolipin) (Ramsey et al., 2006). These families can be divided into two large groups, where TRPC, V, M, and A form the Class I group while polycystins and mucolipins form Class II group of TRP channels (Venkatachalam and Montell, 2007). Here, we will focus on the Class I type expressed in humans.

The first highlights in the discovery of TRP channels originated from observations of a phenotypic characteristic in a mutant strain of the fruit fly *Drosophila* (Cosens and Manning, 1969), where this mutant strain exhibited abnormal responses to light stimuli, indicating potential involvement of specific genes in sensory transduction processes. It was not until 1989 that Montell and Rubin assigned an identity using cDNA libraries obtained from mRNA extracted from the heads of adult *Drosophila*. They managed to identify the mRNA, which they named TRP (Montell and Rubin, 1989). Later, in 1992, they provided the first evidence that TRP proteins function as ion channels (Hardie and Minke, 1992).

TRP channels are tetrameric, non-selective, cation permeating channels structurally similar to voltage-gated potassium channels (Kalia and Swartz, 2013). Each TRP channel protomer comprises six transmembrane segments (S1–6), a pore region between S5 and S6, and intracellularly located N- and C-terminal domains, reviewed in (Cao, 2020; Zhang et al., 2023).

These channels can form homotetramers and/or heterotetramers depending on whether the subunits are identical or present one or more subunits from other TRP channels (Nilius, 2007; Gaudet, 2008; Cabezas-Bratesco et al., 2022). Furthermore, these channels interact with several accessory proteins that can regulate their expression and sub-cellular localization (Moiseenkova-Bell and Wensel, 2011; Cabezas-Bratesco et al., 2022; Cai and Chen, 2023).

Different mechanisms can regulate TRP channels, but in this review, we will focus on regulating the amount of TRP ion channels in the cell surface or trafficking to/from the plasma membrane (Royle and Murrell-Lagnado, 2003). In this context, the localization of an ion channel at the cell surface is determined partly by the balance between its synthesis and degradation, which occurs on a relatively slow time scale, as well as by its constitutive and regulated trafficking mechanisms (Bezzerides et al., 2004; Ambudkar, 2007; Dong et al., 2010; Toro et al., 2011). One of the main regulatory mechanisms of ion channels involves modulating the number of channel molecules expressed at the cell surface, either through the synthesis of new channels or the degradation of existing ones. However, if rapid regulation is needed, it can be achieved by either inserting or removing already synthesized channels stored in intracellular vesicles, which makes trafficking an important mechanism for regulating the activity of ion channels in a rapid fashion (Royle and Murrell-Lagnado, 2003). It is known that the expression of surface proteins can be regulated in various ways, one of which might be due to an increase in protein retention through its interaction with scaffold proteins during or after their insertion into the plasma membrane (Altschuler et al., 2003; Yu et al., 2011; Zhao et al., 2015; Van den Eynde et al., 2021). While the surface expression of ion channels requires the fusion of transporting vesicles mediated by the exocytic pathway, their internalization mechanisms require the activation of endocytic pathways (Royle and Murrell-Lagnado, 2003; Estadella et al., 2020).

Concerning the endocytosis of ion channels, the mechanism relies on various proteins and lipids that are present in specific domains of the plasma membrane, such as clathrin and caveolin, the soluble N-ethylmaleimide-sensitive factor attachment receptor protein (SNARE), or phosphoinositides such as PIP_2_, (Jahn et al., 2003; Laude and Prior, 2004). Different studies have shown that TRP channels traffic to specific cell regions to perform their function in polarized cells such as in the kidney, gastrointestinal tissue, or neurons. However, the mechanisms involved in this specific destination are still unclear for the most part (Altschuler et al., 2003; Yu et al., 2011; Zhao et al., 2015; Van den Eynde et al., 2021). Thus, a better understanding of the differential trafficking of these proteins could explain, for example, how TRPC6 reaches the basolateral and apical membranes of polarized epithelial cells. Also, TRPC3 is present on the apical side and TRPC1 is found mainly on the basolateral membrane (Singh et al., 2001; Bandyopadhyay et al., 2005; Goel et al., 2007).

This review will focus on the mechanisms reported for the trafficking and surface localization of the different class I TRP channels in mammalian cells, providing insights into the proteins involved in their subcellular targeting and trafficking. Moreover, as defects in the localization of ion channels have often been linked to the development of several pathologies, we will also provide an overview of diseases that have been related to the changes in the localization of these channels.

### The TRPC subfamily of ion channels, insights into their trafficking and physiological significance

The TRPC channel subfamily comprises seven members in mammals (TRPC1-7). TRPC2 is a human pseudogene and, therefore, was not considered in this review. Like other members of the TRP channel family (TRPA and TRPV), TRPC channels feature ankyrin repeat sequences within their N-terminal domain. Additionally, like the TRPM subfamily, these channels possess a proline-rich TRP structural domain located in the C-terminal region, close to the channel’s sixth transmembrane segment. On the other hand, in the N-terminal portion are three or four ankyrin repeats depending on the channel and a coiled-coil domain (Philipp et al., 2000; Lepage et al., 2006).

Like other ion channels, TRPC channels can form heterotetramers. For example, the TRPC1 channel can co-assemble with TRPC3-TRPC7 (Wu et al., 2004). TRPC channels can be activated through different mechanisms, such as Gq/11 receptors or receptor tyrosine kinase (RTK) downstream of the phospholipase C pathway (Trebak et al., 2007). Once phospholipase C (PLC) is activated, TRPC3/6/7 channels are activated by diacylglycerol (DAG) independently of protein kinase C (PKC), indicating that DAG mediates their physiological activation (Hofmann et al., 1999; Ma et al., 2000; Venkatachalam et al., 2002). On the other hand, TRPC1/4 and 5 channels are activated by receptor-induced PLC activation and are not responsive to DAG (Hofmann et al., 1999; Venkatachalam et al., 2003).

The function of TRPC channels is determined by their interactions with several proteins that affect their regulation, trafficking, and scaffolding, in addition to their effects on other downstream cellular processes (Ong and Ambudkar, 2011). It has been proposed that these interactions determine their localization and function in specialized plasma membrane microdomains (Ong and Ambudkar, 2011). TRPC channels have various conserved protein-protein interaction motifs in the N- and C-terminal regions, together with those that interact with WW-repeat domains, Cav1-scaffolding domains, PDZ-domains, and lipids such as PIP_2_. Moreover, they have ankyrin repeats and numerous coiled-coiled regions that appear particularly important in the assembly of the channels and their plasma membrane localization. Numerous proteins such as Homer, STIM1, Caveolin, NHERF, and PI3K, interact with specific TRPC channels and regulate their surface expression and channel activity (Table 1). Any alterations in these domains and interactions can result in dysregulation of the surface expression of the channels, contributing to the development of pathologies, reviewed in (Englisch et al., 2022).

**TABLE 1:** Summary of TRPC trafficking interactions.

Channel	Interaction	Effect	References
TRPC1	HOMER	Supports its transfer to the plasma membrane	Yuan et al. (2003)
	β-tubulin; phospholipase C InsP_3_receptor; calmodulin	channel localization by remodeling of the cytoskeleton by depending on the location of microtubules	Clark et al. (2008)
	caveolin-1	prevents the targeting of TRPC1 to the plasma membrane lipid rafts	Sundivakkam et al. (2009)
	IP3R and RhoA	increases the trafficking of TRPC1 by remodeling of the cytoskeleton	Mehta et al. (2003)
	D-amino acid peptide HYD1 (MTI-101)	induces trafficking of TRPC1 to the membrane by induces the formation of a TRPC1/STIM1 complex	Gebhard et al. (2013)
TRPC3	caveolin-1	interrupt the actin cytoskeleton localization of TRPC3 at the plasma membrane	Lockwich et al. (2001)
	EGF	elevation in peripheral TRPC3 localization	Smyth et al. (2006)
	RACK1	serves as a scaffold for localizing TRPC3 in the plasma membrane	Bandyopadhyay et al. (2008)
	Caveolin-1	cooperate with TRPC3 and interrupt the actine cytoskeleton localization of TRPC3 at the plasma membrane	Ma et al. (2000)
			Lockwich et al. (2001)
	1,2bis (2-aminophenoxy)	is necesary for the externalitation of TRPC3	Cayouette et al. (2004), Smyth et al. (2006)
	IP3 receptor	secretion-like trafficking of channels to the plasma membrane	Putney et al. (2004)
	Gd3^+^	binds to TRPC3 channles at and exterior site, causing incapacity of bound channels to be proprely internalizated	Smyth et al. (2006)
	SNARE	cytoskeleton disruption, generating effects on constitutive trafficking of TRPC3 controlling translocation	Singh et al. (2004)
	VAMP-2	involved in fusion of the vesicles to the plasma membrane and produce the insertion of TRPC3 channels	Singh et al. (2004), Van Rossum et al. (2000)
TRPC4/5	NHERF	facilitates association of TRPC4 and TRPC5 with PLCβ and increase their surface expression	Mery et al. (2002)
	EGF	activation of tirosine kinase and produce the phosporilatyon of TRPC4 and boots the exocytic attachment into the plasma membrane	Mery et al. (2002)
	Ezrin	regulate the vesicular trafficking of TRPC4	Mery et al. (2002)
	stathmin 2	trafficking to the growthone is facilitated	Greka et al. (2003)
	PI4,5P2	involved in the interaction with the membrane/targeting domain at the N-ter of TRPC4/5 heteromes	Myeong et al. (2014)
	EBP50	attached TRPC4 to the cytoskeleton and prevent its internatilzation	Mery et al. (2002)
	PI3-kinase (PI3K) Rac1, PI4P-5 kinase	Integration of the channel into the plasma membrane	Bezzerides et al. (2004)
	E3KARP	cell surface delivery and translocation of TRPC4-bearing vesicles	Mery et al. (2002)
TRPC6	Munc13 family	fusion of TRPC6-containing vesicles with the plasma membrane	Xie et al. (2020)
	Human myxovirus resistance protein 1 (MxA)	increase the activity of TRPC6	Lussier et al. (2005)
	caveolin 1/2	vesicular trafficking	Cayouette et al. (2004)
	PLC-γ1	increased expression in the cell surface	Kanda et al. (2011)
	nephrin	protein binds directly to TRPC6 after phosphorylation of the Y284 residueand suppresses its translocation by interfering with TRPC6–PLC-γ1 binding	Kanda et al. (2011)
TRPC7	Human myxovirus resistance protein 1 (MxA)	enhances channel activity by regulating trafficking of these proteins to the plasma membrane	Lussier et al. (2005)

### TRPC1

TRPC1 is expressed in a large range of cell types and tissues. TRPC1 was originally found in the fetal brain, liver, and kidney tissues, as well as the adult heart, testes, ovaries, and brain (Wes et al., 1995). After these first descriptions, different groups have found the channel to be broadly expressed in mammalian tissues (Vazquez et al., 2004a; Beech, 2005; Ramsey et al., 2006; Ambudkar, 2013; Dietrich et al., 2014). Up to this point, TRPC1 performs a role in intracellular Na^+^ and Ca^2+^ homeostasis and although its function is not entirely clear (Hoth and Penner, 1992; Feske et al., 2006; Vig et al., 2006; Dietrich et al., 2014).

The trafficking of TRPC1 channels is controlled by several mechanisms involving interactions with lipid modifications, scaffolding proteins, and post-translational modifications (Ferrandiz-Huertas et al., 2014a). For instance, the interaction of TRPC1 with the scaffolding protein Homer has been shown to support its transfer to the plasma membrane, whereas palmitoylation of TRPC1 channels is involved in regulating their permanence and localization in the plasma membrane (Kiselyov et al., 1998; Torihashi et al., 2002; Yuan et al., 2003; Ambudkar et al., 2004). Moreover, TRPC1 localization is regulated by remodeling of the cytoskeleton through interaction with several proteins, including β-tubulin, IP_3_ receptor, calmodulin (CaM), PLC, and several other proteins (Clark et al., 2008). TRPC1 interacts with caveolin-1 through the C- and N-terminal domains. This interaction seems relevant in assembling a signaling complex localized in lipid rafts, important for regulating Ca^2+^ signaling (Sundivakkam et al., 2009).

The expression of an N terminal-truncated TRPC1 or a dominant-negative caveolin mutant prevents the TRPC1 trafficking to the plasma membrane affecting Ca^2+^ influx associated with direct activation of TRPC1 by agonists or passive store depletion. Additionally, in endothelial cells, trafficking of TRPC1 to the membrane occurs following thrombin stimulation (Mehta et al., 2003). Following activation, TRPC1 assembles in a complex with the IP_3_R and Ras homolog family member A (RhoA), a protein essential for the remodeling of the cytoskeleton (Liu et al., 2000; Lockwich et al., 2000; Brazer et al., 2003; Isshiki and Anderson, 2003; Mehta et al., 2003; Ambudkar et al., 2004; Brownlow et al., 2004; Bollimuntha et al., 2005). Impairments in TRPC1 channel trafficking have been related to various diseases, including cardiac hypertrophy. In ventricular myocytes, TRPC1 colocalized with sarcoplasmic/endoplasmic reticulum Ca^2+^-ATPase 2 (SERCA2), an established marker of sarcoplasmic reticulum (SR), and sarcomeric α-actinin, which is a marker of the Z-line of the cardiomyocytes (Hu et al., 2020).

In this context, mutations in the TRPC1 gene have been identified in patients with familial essential hypertension (Jiang et al., 2014). Moreover, the abdominal aortic-banded model (AAB) in animals generates an upregulation of TRPC1 expression and an increased store-operated calcium entry (SOCE) in hypertrophied cardiomyocytes. In contrast, the knockdown of TRPC1 decreased SOCE. In this study, cardiomyocytes were treated with d-amino acid peptide HYD1 (MTI-101), which induces trafficking of TRPC1 to the membrane. Co-immunoprecipitation studies indicate that MTI-101 treatment induces the formation of a TRPC1- stromal interaction molecule 1 (STIM1) complex (Gebhard et al., 2013). This influx of Ca^2+^ activates the SOCE pathway and allows TRPC1 trafficking and insertion into the plasma membrane. Similar observations were made in NCI-H929 and U266 myeloma cell lines where aberrant trafficking of TRPC1 channels has been linked to the growth and metastasis of tumor cells (Gebhard et al., 2013; Myeong et al., 2018; Elzamzamy et al., 2020; Elzamzamy et al., 2021). Overall, the proper trafficking of TRPC1 channels to the membrane is crucial for its function in SOCE, and defects in trafficking can have profound effects on cellular physiology and human health.

### TRPC3

TRPC3 is a non-selective cation permeable channel that performs a critical role in several physiological activities, including cardiac hypertrophy, vascular smooth muscle contraction, and insulin secretion among others (Vazquez et al., 2004a). Various factors, including post-translational modifications and the activity of molecular chaperones through protein-protein interactions control this process (Li et al., 1999; Gonzalez-Cobos and Trebak, 2010). The proper localization of TRPC3 channels to the plasma membrane is vital for their function (Xia et al., 2015; Oda et al., 2017; Fan et al., 2018).

TRPC3 can be found assembled in a multimeric protein complex with key Ca^2+^ signaling proteins such as PLCβ, G alpha-q (Gαq) subunit of heterotrimeric G protein (Gαq/11), Inositol trisphosphate receptor (IP3Rs), CaM and PLCγ (Kiselyov et al., 1998; Lockwich et al., 2001; Zhang et al., 2001; Patterson et al., 2002; Wedel et al., 2003). However, the precise mechanisms governing TRPC3 trafficking to the plasma membrane remain unresolved.

Singh and colleagues proposed that agonist-induced activation of TRPCs also triggers exocytotic trafficking of the channel. They observed that carbachol (CCh) induces a vesicle-associated membrane protein 2 (VAMP-2)-dependent increase in the level of TRPC3 in the plasma membrane, independently of intracellular calcium concentration. TRPC3-containing vesicles were found to be positioned immediately beneath the plasma membrane, likely pre-docked to specific sites through the action of docking and scaffolding proteins. Moreover, they suggested that the surface expression of TRPC3 is regulated by both a recycling-type trafficking mechanism and a regulated event stimulated by CCh (Singh et al., 2004). Furthermore, in 2008, Bandyopadhyay and colleagues reported that the receptor for activated C-kinase-1 (RACK1) serves as a scaffold for localizing TRPC3 in the plasma membrane of cells. This interaction plays a crucial role in recruiting the channel into an IP3R-associated signaling complex and facilitating its insertion into the cell surface membrane upon stimulation by agonists (Bandyopadhyay et al., 2008).

In this context, CCh-induced exocytic trafficking of TRPC3 has been probed by surface biotinylation labeling showing that channel externalization occurs in the presence of the calcium chelator 1,2-bis (2- aminophenoxy) ethane-N,N,N,N-tetraacetic acid (BAPTA), reducing the possibility of a secondary effect by the Ca^2+^ mobilizing activity of the agonists (Cayouette et al., 2004; Smyth et al., 2006). On the other hand, several studies propose that TRPC3 trafficking is not mandatory for channel activation. They hypothesize that channel activation by second messengers, including DAG or conformational coupling of TRPC channels with the IP_3_R, could lead to secretion-like trafficking of channels to the plasma membrane (Vazquez et al., 2004b; Putney et al., 2004). Moreover, Smyth and colleagues demonstrated that Gd^3+^ binds to TRPC3 channels at an extracellular site, causing impaired internalization. Gd^3+^ effectively “locks” TRPC3 in the plasma membrane, favoring constitutive exocytosis over endocytosis and resulting in a swift accumulation of TRPC3 in the membrane. Furthermore, they observed an elevation in peripheral TRPC3 localization induced by epidermal growth factor (EGF), as assessed by total internal reflection fluorescence (TIRF) microscopy. This suggests that EGF may similarly stabilize TRPC3 in the plasma membrane by impeding its endocytosis, although the precise mechanism remains unclear (Smyth et al., 2006). Nonetheless, it is increasingly evident that the constitutive cycling of plasma membrane proteins is a fundamental mechanism for regulating surface membrane proteins (Royle and Murrell-Lagnado, 2003). TRPC3 also interacts with caveolin-1, a marker for caveolae, and disruption of the actin cytoskeleton deregulates the localization of caveolin-1 and TRPC3 at the plasma membrane (Ma et al., 2000; Lockwich et al., 2001).

Additionally, it has been reported that the N-terminal of TRPC3, specifically in amino acids 123-221, interacts directly with VAMP-2. VAMP-2 is located in the membrane of intracellular trafficking vesicles and mediates the fusion of vesicles to the plasma membrane, promoting the insertion of TRPC3 in the plasma membrane (van Rossum et al., 2000; Singh et al., 2004). Up to this point, the involvement of SNARE proteins in regulating TRPC3 localization at the plasma membrane is still not completely clear.

Importantly, atypical trafficking of TRPC3 channels can lead to diseases, including cardiac hypertrophy, hypertension, and Alzheimer’s disease (Tano et al., 2010; Smedlund et al., 2012; Tiapko and Groschner, 2018). It has been described that in cardiac hypertrophy, excessive trafficking of TRPC3 channels can lead to enhanced Ca^2+^ influx and stimulation of downstream signaling pathways like nuclear factor of activated T-cells (NFAT) and mammalian target of rapamycin (mTOR), leading to the development of hypertrophy (Brenner and Dolmetsch, 2007). These channels have been associated with regulating blood pressure, and changes in TRPC3 trafficking have been linked to pulmonary hypertension (Tano et al., 2010). In the brain, it is involved in the pathogenesis of Alzheimer’s disease, where Aβ peptides, which are linked to the development of Alzheimer’s disease, correlate with TRPC3-dependent calcium influx, leading to neurotoxicity (Montecinos-Oliva et al., 2014; Wang L. et al., 2017).

### TRPC4/5

TRPC4/5 channels are ubiquitously expressed in various tissues. They play a role in calcium signaling and ion homeostasis in various relevant physiological processes, including taste and vision, microvascular permeability, vasorelaxation, gastrointestinal motility, and neurotransmitter release (Fujita et al., 2017; Kim et al., 2019). Like many other membrane proteins, TRPC4 is synthesized in the ER, undergoing post-translational modifications, including glycosylation and phosphorylation. Then it is shipped to the Golgi apparatus, where it is further modified and sorted for delivery to the plasma membrane (Duan et al., 2018). Moreover, TRPC channels are particularly sensitive to mutations and deletions. These modifications may lead to reduced trafficking to the plasma membrane and their retention in the ER or Golgi (Xu et al., 2001; van de Graaf et al., 2003; Wedel et al., 2003; Andrade et al., 2005).

Additionally, two different studies showed that TRPC4 and TRPC5 interact with PDZ-domain-containing proteins such as the Na^+^/H^+^ exchanger regulatory factor (NHERF) and the gap junction protein zona occludens 1 (ZO-1) via their C-terminal PDZ-interacting domains (Weinman et al., 1995; Tang et al., 2000). The interface with NHERF facilitates the association of TRPC4 and TRPC5 with PLCβ, which, in turn, positively regulates their surface expression (Mery et al., 2002). Moreover, cellular stimulation by EGF activates protein tyrosine kinases, leading to the specific phosphorylation of two tyrosine residues located at the C-terminal end of TRPC4 (Mery et al., 2002). Such post-translational modification boosts the interaction of TRPC4 and NHERF and the plasma membrane expression of the channel (Mery et al., 2002).

TRPC4 is regulated by the growth factor receptor signaling proteins, such as ezrin, in addition to NHERF binding proteins, which regulate the vesicular trafficking of TRPC4 (Mery et al., 2002). In this context, it has been observed that TRPC5 is trafficked to certain sites in hippocampal neurons and that TRPC5 homomers are found in growth cones (Greka et al., 2003). Trafficking of TRPC5 to the growth cone is facilitated *via* attaching to the exocytic protein stathmin 2 (a protein associated with ALS), where Ca^2+^ influx through the channel prevents the extension of growth cones. On the other hand, SNARE proteins interact with the TRPC5 trafficking complex (Greka et al., 2003). TRPC5 channels are localized in vesicles, and fast trafficking of these vesicles to the plasma membrane is triggered in response to stimulation with EGF and nerve growth factor (NGF) and a faint response to brain-derived neurotrophic factor (BDNF) and insulin-like growth factor-1 (IGF-1). Integration of the channel into the plasma membrane also involves Phosphoinositide 3-kinase (PI3K) and the GTPase Rac Family Small GTPase 1 (Rac1), as well as PI4P-5 kinase (Bezzerides et al., 2004). Additionally, TRPC5-including vesicles seem to be retained in a subplasma membrane region from where they are rapidly recruited to the membrane (Ambudkar, 2013).

Mery *et al.* (2002) showed that the proximal N-terminal region of TRPC4 (amino acids between 23 and 29 in the mice ortholog) is vital for membrane insertion of TRPC4 channels. Altering this domain might cause a decrease in trafficking to the plasma membrane by preventing either the appropriate folding or heterotetrameric assembly with wild-type TRPC4 (Mery et al., 2002). Myeong *et al.* (2014) demonstrated that the N- and C-terminal regions interact with each other to make a tetrameric structure and that PI(4,5)P_2_ is a strong candidate involved in the interaction with the membrane-targeting domain at the N terminus of TRPC4/5 heteromers. They reported that the N terminal (amino acids 98–124) and C-terminal (amino acids 700–728) were necessary for the tetrameric assembly of TRPC4 (Myeong et al., 2014; Hong et al., 2015). The interaction between the scaffold ERM-binding phosphoprotein 50 (EBP50) (the NHERF human ortholog) and the membrane-cytoskeleton adaptors can attach TRPC4 to the cytoskeleton and prevent its internalization. NHE3 kinase A regulatory protein (E3KARP), a member of the ezrin/radixin/moesin (ERM) family, plays an active role in the cell surface delivery of TRPC4 by enabling the translocation of TRPC4-bearing vesicles from the cortical actin layer to the plasma membrane. Removal of the last three C-terminal amino acids in TRPC4 blocks the interaction with EBP50 and alters the channel’s localization and surface expression (Mery et al., 2002). In the same study, the authors suggested that TRPC4 can reach the submembranous compartment but is not delivered to the cell surface (Mery et al., 2002).

Dysregulation of TRPC4/5 trafficking and/or function can lead to various diseases (Myeong et al., 2014) in the brain (Mori et al., 1998; Fowler et al., 2007), peripheral sensory neurons (Wu et al., 2008), and gastrointestinal organs. Moreover, a few articles have implicated TRPC4 in ocular diseases, including retinitis pigmentosa and glaucoma (Yang et al., 2005; Reinach et al., 2015; Yang et al., 2022). In retinitis pigmentosa, mutations in TRPC4 result in abnormal protein trafficking to the plasma membrane, leading to photoreceptor cell death and vision loss. In glaucoma, the dysregulation of TRPC4 trafficking is thought to contribute to the death of retinal ganglion cells, which leads to optic nerve damage and vision loss (Yang et al., 2005; Reinach et al., 2015; Yang et al., 2022).

TRPC4 is also associated with several cardiovascular diseases, including cardiac hypertrophy, heart failure, and hypertension. TRPC4 plays a crucial role in regulating the heart’s electrical activity, and abnormal trafficking of TRPC4 can lead to dysregulation of these processes. Mutations in the TRPC4 gene have been associated with atrial fibrillation, Brugada syndrome, and long QT syndrome, whereas TRPC5 has been linked to the development of cardiac hypertrophy and heart failure, as well as pulmonary hypertension (Watanabe et al., 2008; Camacho et al., 2015; Lüscher, 2015; Du et al., 2021). TRPC5 regulates Ca^2+^ signaling and vascular smooth muscle tone, crucial cardiovascular physiology processes. Impaired TRPC4 trafficking can lead to calcium overload in cardiomyocytes, which dysregulates their function and can lead to heart dysfunction. TRPC4 is also involved in regulating vascular smooth muscle tone, and alterations in its activity can lead to increased blood pressure and hypertension (Watanabe et al., 2008; Camacho et al., 2015; Lüscher, 2015; Du et al., 2021).

TRPC4 has been implicated in several neurological disorders, including multiple sclerosis and Parkinson’s disease, due to its involvement in the regulation of calcium signaling and neuronal excitability (Balbuena et al., 2012; Wang L.-K. et al., 2017; Enders et al., 2020). On the other hand, TRPC5 has been linked to depression, anxiety, and drug addiction (Yang et al., 2005; Nazıroglu and Demirdas, 2015; Yang et al., 2015; Sharma and Hopkins, 2019). Studies have shown that TRPC5 regulates the release of neurotransmitters and modulates synaptic plasticity, which are important processes in the pathogenesis of these disorders (Puram et al., 2011; He et al., 2012). Recent studies have shown that TRPC4 regulates the volume of airway epithelial cells, and its dysfunction may contribute to the pathogenesis of cystic fibrosis (Müller et al., 2022). TRPC5 is predominantly expressed in the renal system and has been linked to the pathogenesis of several renal diseases, including focal segmental glomerulosclerosis (FSGS), diabetic nephropathy, and polycystic kidney disease (Zhou et al., 2017; Yu et al., 2019; Walsh et al., 2021).

### TRPC6

Several factors can affect the TRPC6 expression on the plasma membrane of channels in non-excitable cells. For instance, the stimulation of endogenous muscarinic receptors with CCh and the depletion of intracellular calcium stores generate the translocation of TRPC6 toward the plasma membrane (Cayouette et al., 2004). There is still no clear evidence of the participation of SNARE proteins in the trafficking of TRPC6. It has been reported that the Human myxovirus resistance protein 1 (MxA), closely related to the membrane-remodeling GTPase dynamin, interacts with TRPC6 (Lussier et al., 2005). Among the proteins involved in TRPC6 trafficking induced by DAG stimulation, the mammalian uncoordinated-13 (Munc13) family of proteins participates in the fusion of TRPC6-containing vesicles with the plasma membrane, providing insights into the DAG-regulated vesicle fusion mechanism (Xie et al., 2020).

Regarding the glycosylation of TRPC6, it has been described that it is glycosylated in residues N474 and N561. The removal of glycosylation in residue N561 is sufficient to convert it into a constitutively active channel, while trafficking and surface expression remain unaffected in glycosylation-deficient mutants (Dietrich et al., 2003; Talbot et al., 2019).

As mentioned above, TRPC6 can form heterotetramers with other members of the TRPC channel family. Three independent studies observed that once activated, TRPC6 is translocated to the plasma membrane along with TRPC3 (Cayouette et al., 2004; Singh et al., 2004; Kim et al., 2006). In another study, HEK293 cells were stimulated with CCh, resulting in the translocation of TRPC6 in the caveolae, which are closely linked to vesicular traffic (Cayouette et al., 2004; Cayouette and Boulay, 2007). Interestingly, this translocation mechanism is independent of intracellular calcium increases (Stojilkovic et al., 2005).

Moreover, another mechanism involved in TRPC6 trafficking was reported by Kanda *et al.* (2011), who demonstrated that phosphorylation of a single residue (Y284 in the mice ortholog) is required for channel surface expression. Moreover, a tyrosine to phenylalanine mutation (Y284F) showed decreased expression on the cell surface of both HEK293T cells podocyte cultures, suggesting that the residue is necessary for PLC-γ1 binding (Kanda et al., 2011). In addition, the authors observed that in podocytes, the nephrin protein binds directly to TRPC6 after phosphorylation of Y284, suggesting that a naturally occurring mutation in this position might be associated with focal segmental glomerulosclerosis disease (FSGS) (Kanda et al., 2011). Coincidently, Hagmann and colleagues (2018) have proposed that phosphorylation at S14 in mice TRPC6 enhances channel conductance by increasing the membrane expression of TRPC6 (Hagmann et al., 2018).

### TRPC7

The TRPC7 channel is the most recently identified member of the TRPC family, and little is known about the mechanisms governing its trafficking to the plasma membrane. Additionally, there is not much information about naturally occurring mutations that can affect the channel’s expression and/or trafficking. Lussier *et al.* (2005) demonstrated through different approaches, including GST-pull-down and co-immunoprecipitation assays, that MxA interacts with the second ankyrin-like repeat domain of TRPC7 and other TRP channels. It has been proposed that MxA enhances channel activity by regulating trafficking to the plasma membrane (Lussier et al., 2005). However, this functional role seen in other TRP channels has not been tested on TRPC7 yet.

**FIGURE 1:**
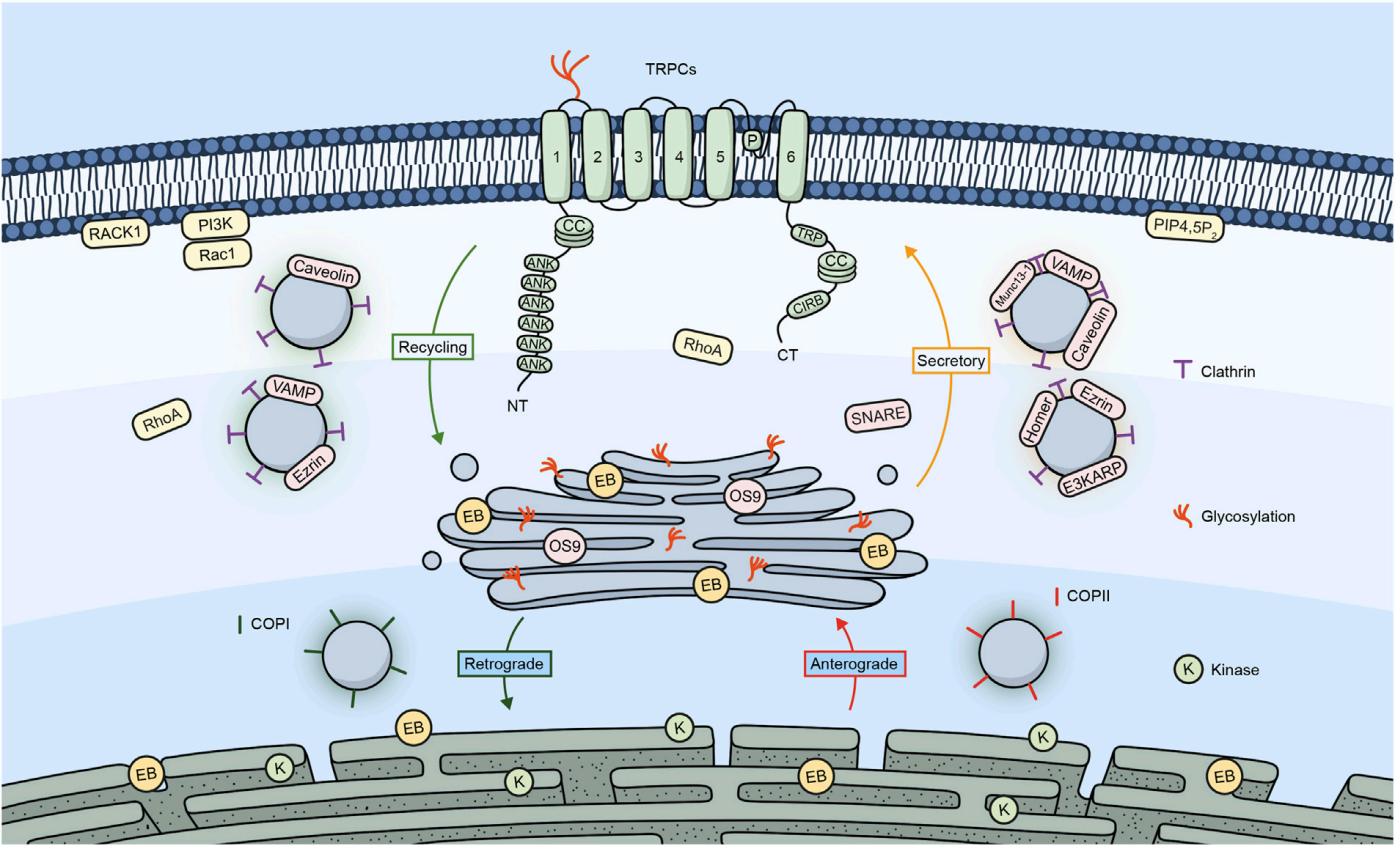
TRPC channels and their trafficking regulators. Embedded in the plasma membrane is a monomeric subunit representing the various domains and regions in TRPC channels. These include ANK (ankyrin repeats), CC (coiled-coil), TRP, which refers to the TRP box common to all TRP channels and a calmodulin, and IP_3_R binding site (CIRB). The diagram shows a summary of the different regulators involved in the trafficking of TRPC channels.

Figure 1 shows a summary of the different proteins/molecules involved in the regulation of the trafficking of TRPC channels to the plasma membrane as were described in this section of the review.

## The TRPM subfamily of ion channels, insights into their trafficking and physiological significance

The human TRPM subfamily is the largest among those belonging to the superfamily of TRP channels and was named after the founding member Melastatin (TRPM1) (Fleig and Penner, 2004). Since the first description and cloning of TRPM1 in 1998 (Duncan et al., 1998), seven more members have been added to this subfamily, which can be grouped into four pairs based on their structural similarity (TRPM1/TRPM3; TRPM2/TRPM8; TRPM4/TRPM5; and TRPM6/TRPM7) (Cohen and Moiseenkova-Bell, 2014). As the rest of the TRP channels, TRPMs are intrinsic membrane proteins with six transmembrane domains that form functional tetramers. TRPMs are widely expressed in the human body. Most TRPMs are Ca^2+^-permeable cation channels, except for TRPM4 and TRPM5, which are Ca^2+^-activated, non-selective, monovalent permeable cation channels (Chen et al., 2019; Yamaguchi et al., 2019). The members of the TRPM subfamily of ion channels are characterized by their polymodal nature of activation as they are regulated by different stimuli such as voltage, temperature, and the binding of ions, lipids, or other ligands (Ramon et al., 2007; Diaz-Franulic et al., 2021).

The TRPM subfamily has garnered greater attention due to its reported involvement in several physiological processes, such as taste transduction (Liu and Liman, 2003), temperature sensing (McKemy et al., 2002; Brauchi et al., 2004), synaptogenesis and neurite outgrowth (Abumaria et al., 2019), cell death (McNulty and Fonfria, 2005), and regulation of vasculature (McNulty and Fonfria, 2005). Moreover, the abnormal expression and/or function of TRPM channels has also been involved in a plethora of pathological processes such as cancer (Hantute-Ghesquier et al., 2018; Saldías et al., 2021), neurological disorders (Sita et al., 2018; Lavanderos et al., 2020), kidney disorders (Hsu et al., 2007), and others (Jimenez et al., 2020). All this poses TRPM channels and their regulation as important therapeutic targets.

In the following section, we will discuss the main mechanisms involved in the trafficking of TRPM channels and how the dysregulation of this process might trigger different pathologies. Like TRPC channels, the trafficking of TRPM channels is regulated by specific protein interactions summarized in Table 2.

**TABLE 2:** Summary of TRPM trafficking interactions.

Channel	Interaction	Effect	References
TRPM1	glutamate receptor 6 (mGluR6)	correct localization of TRPM1	Agosto et al. (2021), Xu et al. (2012)
	nyctalopin	forms complexes with TRPM1 and is necessary for the correct localization of the channel	Cao et al. (2011)
	LRIT3	scaffold for the formation of the complex nyctalopin/mGluR6 for the correct localization	Hasan et al. (2019), Neuillé et al. (2015)
TRPM2	reactive oxygen species (ROS)	increases of TRPM2 in the plasma membrane	Ali et al. (2021), Kheradpezhouh et al. (2018)
TRPM3	exon 13 of the trpm3 gene	regulates channel insertion in the plasma membrane	Frühwald et al. (2012)
	activating transcription factor 4 (ATF4)	regulates KIF17 kinesin-mediated TRPM3 trafficking	Xie et al. (2021)
TRPM4	14-3-3γ protein	promotes membrane targeting of the channel	Cho et al. (2014)
	EB1 and EB2 proteins	increase expression of the channel in the plasma membrane and prevents retention in the ER	Blanco et al. (2019)
	reactive oxygen species (ROS)	lowers the channel density in mouse cortical collecting duct cells	Wu et al. (2016a)
TRPM6	metformin drug	increases/decreases TRPM6 expression in the plasma membrane depending on exposure time	Bouras et al. (2020)
	EGF receptor/src/Rac1	increase in TRPM6 trafficking to the plasma membrane	Thebault et al. (2009)
	TNFα	regulate TRPM6 trafficking to the plasma membrane	Furukawa et al. (2017)
	TRPM7	necessary for the correct trafficking of TRPM6 to the plasma membrane	Schmitz et al. (2005), Shari et al. (2010)
TRPM7	14-3-3θ chaperon protein	regulates channel trafficking facilitating autophosphorilation residues in the channel	Cai et al. (2018)
	TRPM6	alters TRPM7 trafficking	Brandao et al. (2014)
TRPM8	brefeldin A-resistance factor 1 (GBF1)	regulates trafficking of TRPM8 along the cold sensitive peripheral axons	Cornejo et al. (2020)
	TRP channel–associated factors (TCAFs)	promotes TRPM8 trafficking to the plasma membrane	Gkika et al. (2015)
	Vesicle-associated membrane protein 7 (VAMP7)	promotes the trafficking of TRPM8 to the cell surface	Ghosh et al. (2016)

### TRPM1

TRPM1 received the name Melastatin due to its initial discovery in the B16 mouse melanoma cell line (Duncan et al., 1998). TRPM1 expression is not as ubiquitous as with other TRPM channels. Its expression has been reported in the heart, brain, skin, and retina (Fonfria et al., 2006; Oancea and Wicks, 2011). As mentioned before, TRPM1 is involved in Melanoma progression, where expression of TRPM1 inversely correlates with tumor aggressiveness and tumor thickness (Deeds et al., 2000; Guo et al., 2012). It has also been linked to disease-free survival in patients with melanoma (Brozyna et al., 2017), suggesting that TRPM1 may function as a metastatic suppressor. Additionally, it has been reported that TRPM1 is the ion channel that initiates the ON visual pathway in vertebrate vision in the eye (Koike et al., 2010; Nakamura et al., 2010; Iosifidis et al., 2022). TRPM1 trafficking mechanisms have not been fully described yet, but a few reports have suggested possible mechanisms. Nakamura and colleagues showed in ON bipolar cells that mutations R624C and F1075S lead to lower expression in the dendritic tips and mislocalization of TRPM1, which translates to stationary night blindness in patients. These results suggest that these residues are important for the protein trafficking of TRPM1 (Nakamura et al., 2010). Interestingly, the metabotropic glutamate receptor 6 (mGluR6), specifically its C-terminal region, appears to have an important role in the correct localization of TRPM1 to the dendritic tips of depolarizing bipolar cells, as the mGluR6 knockout and other mutants show reduced to no expression of TRPM1 in this region (Xu et al., 2012; Agosto et al., 2021). Moreover, it has been demonstrated that nyctalopin also forms complexes with TRPM1 and is necessary for the correct channel localization (Cao et al., 2011). Additionally, another molecule that has been involved in the trafficking of TRPM1 and the formation of its complex with nyctalopin and mGluR6 is Leucine Rich Repeat, Ig-Like And Transmembrane Domains 3 (LRIT3), which might serve as a scaffold for the formation of this complex since the knockout mouse model for this protein showed no localization of TRPM1 at the dendritic spines of ON bipolar cells (Neuillé et al., 2015; Hasan et al., 2019). These reports suggest that mutations in the interacting residues of TRPM1 with proteins might result in channel trafficking defects.

### TRPM2

TRPM2 is a ligand-gated Ca^2+^-permeable non-selective cationic channel. Despite having a structure that is very similar to the rest of the TRPM subfamily, TRPM2 possesses a C-terminal Nudix hydrolase 9 homologue (NUDT9-H) domain that serves as a binding site for cytosolic adenosine diphosphate ribose (ADPR) (Huang et al., 2018). The gating mechanism of TRPM2 channels seems to require simultaneous co-activation by Calcium (Csanády and Törocsik, 2009), PIP_2_ (Tóth and Csanády, 2012), and ADPR (Perraud et al., 2001; Hara et al., 2002). Although TRPM2 contains this NUDT9-H domain, it has little to no ADPR-degrading ability and would only act as a regulatory ligand binding site (Iordanov et al., 2016). In terms of expression, TRPM2 is nearly ubiquitous in all human tissues and, thus, has been implicated in regulating several physiological and pathological processes (Belrose and Jackson, 2018). TRPM2 plays an important role in migration and chemokine release (Yamamoto et al., 2008). TRPM2 modulates cell migration and invasion in neuroblastoma, and its expression has been associated with poor patient prognosis (Bao et al., 2022), whereas it regulates autophagy during replication of the Hepatitis B virus (Chen et al., 2022). Moreover, it has also been involved in regulating cell death in several cell models (Malko et al., 2019; Zielinska et al., 2021) and as a potential therapeutic target for treating neurological diseases (Sita et al., 2018; Malko et al., 2019).

About trafficking, the most studied regulatory mechanism of TRPM2 is the effect that reactive oxygen species (ROS) have over its localization. Reports have shown that ROS increase TRPM2 activity (Ali et al., 2021) and treatments with either H_2_O_2_ or the non-opioid analgesic acetaminophen (both leading to the production of ROS) increase TRPM2 in the plasma membrane (Kheradpezhouh et al., 2018). Moreover, Mei and Jiang reported that although they saw no effect for the N-terminal coiled-coil domain of TRPM2 in the trafficking of the channel, they could not discard a minor contribution of this domain to the transport of the channel (Mei and Jiang, 2009). Additionally, the expression of a short splicing variant of TRPM2 decreased the channel’s activity, mostly by decreasing the trafficking of the full-length channel to the plasma membrane (Yamamoto et al., 2019).

### TRPM3

TRPM3 is a Ca^2+^ permeable, non-selective cation channel activated by heat and chemical activators which, interestingly, shares certain homology with the heat sensitive TRPV channels (Vriens et al., 2011). As with other TRPM channels, TRPM3 is expressed in a wide variety of tissues in the body (Held et al., 2015), with a relatively higher expression in the brain, spinal cord, sensory neurons, pituitary, kidney and eye (Fonfria et al., 2006; Shaham et al., 2013; Oberwinkler and Philipp, 2014). TRPM3 plays an important role in pancreatic β-cells, where it regulates zinc uptake (Wagner et al., 2010), and in the eye, where its mutations lead to cataracts and glaucoma (Bennett et al., 2014; Zhou et al., 2021). However, its most reported role is the one it plays as a nociceptor in detecting noxious heat and developing inflammatory heat hypersensitivity (Held et al., 2015; Mickle et al., 2015; Held and Tóth, 2021).

The mechanisms involved in TRPM3 trafficking are not well understood. However, we have some information from the few works that have addressed the topic. It has been reported that an 18 amino acid sequence encoded by exon 13 of the *trpm3* gene regulates channel insertion in the plasma membrane and is key for sustaining TRPM3 activity (Frühwald et al., 2012). Moreover, it has been recently proposed that mutations in the N-terminal part of the protein, specifically the L769V mutation, affect trafficking to the plasma membrane and result in a non-functional channel (Burglen et al., 2023). Additionally, it has been reported that the activating transcription factor 4 (ATF4), a member of the CREB family of proteins, regulates kinesin family member 17 (KIF17) -mediated TRPM3 trafficking by interacting with KIF17-TRPM3 complex and modulating dorsal root ganglia (DRG) neuron response to heat stimuli (Xie et al., 2021).

### TRPM4

TRPM4 differs from most other TRP channels by not being permeable to Ca^2+^ but only to monovalent cations (showing a high selectivity for Na^+^). However, one of its main features is that intracellular Ca^2+^ can directly activate it (Launay et al., 2002; Ullrich et al., 2005). TRPM4 is expressed widely throughout the different body tissues, although several reports have shown a higher expression level in the colon, heart, and prostate (Launay et al., 2002; Fonfria et al., 2006; Syam et al., 2016; Borgstrom et al., 2021). Moreover, TRPM4 expression has been reported in several other cell types, such as immune cells, neuronal tissue, and pancreatic β-cells (Cheng et al., 2007; Barbet et al., 2008; Schattling et al., 2012). This wide expression pattern underlines the important role this channel might play in several physiological and pathological processes. TRPM4 has been reported to play a role in pathologies such as different types of cancer (Rivas et al., 2020; Borgstrom et al., 2021; Wang et al., 2022), and cardiovascular (Stallmeyer et al., 2012; Syam et al., 2016; Wang et al., 2018) and immune system-associated diseases (Barbet et al., 2008; Schattling et al., 2012; Serafini et al., 2012).

Trafficking mechanisms of TRPM4, unlike other TRPM channels, have been more widely studied in recent years. Cho et al. (2014) demonstrated that membrane targeting of the channel is regulated by interaction with the 14-3-3γ protein through the N-terminal region of TRPM4 and that S88 in the human ortholog, which might be a target of phosphorylation, is key for 14-3-3γ binding and anterograde trafficking of TRPM4 (Cho et al., 2014). On the other hand, in patients with human progressive familial heart block type I, the E7K mutation at the N-terminal domain caused attenuated deSUMOylation of the channel, which impaired channel endocytosis and led to an increased activity of TRPM4 due to an elevated concentration of the channel in the plasma membrane (Kruse et al., 2009). Interestingly, several other mutations showed a similar effect in patients with isolated cardiac conduction. Mutations R164W, A432T, and G844D enhance TRPM4 current density due to increased channel abundance in the membrane caused by SUMOylation defects and impaired endocytosis (Liu et al., 2010). Moreover, in patients with congenital or childhood atrioventricular block, mutations A432T and A432T/G582S showed decreased protein expression at the cell membrane, whereas G582S alone showed increased membrane expression of the channel (Syam et al., 2016). Although N-glycosylation also plays an important role in the surface expression and trafficking of ion channels, its role in TRPM4 trafficking is somehow controversial. Several reports showed that the N988 residue in rats and N992/N932 residues in humans are glycosylation sites for TRPM4, but these mutations did not affect the surface expression of the channel but rather modulated its stability (Woo et al., 2013; Syam et al., 2014). Interestingly, phosphorylation also appears to have a role in TRPM4 trafficking. Phosphorylation of S839 is necessary for basolateral localization of TRPM4 in epithelial cells (Cerda et al., 2015). Other mutations have been reported to impact TRPM4 trafficking without an apparent change in the electrophysiological properties of the channel, such as those described in Brugada syndrome, where P779R and K914X showed a decreased membrane expression whereas T873I and L1075P showed an increase in membrane localization of TRPM4 (Liu et al., 2013). Another mechanism reported for TRPM4 anterograde trafficking is its interaction with end-binding (EB) proteins. Blanco *et al.* (2019) showed that mutations in the EB binding motif in TRPM4 prevented its interaction with EB proteins, which led to a reduced expression of the channel in the plasma membrane and retention in the ER (Blanco et al., 2019). Interestingly, TRPM4 localization in the plasma membrane is also modulated by ROS, given that hydrogen peroxide treatment lowers the channel density in mouse cortical collecting duct cells (Wu M. et al., 2016).

### TRPM5

TRPM5, like TRPM4, is a nonselective monovalent cation channel impermeable to divalent cations activated by Ca^2+^ and has a high permeability for Na^+^ (Prawitt et al., 2003). However, unlike TRPM4, which is widely expressed in several tissues and organs, TRPM5 expression is more limited to highly specialized cells. For example, high expression of the channel has been reported in β cells from pancreatic islets of Langerhans, where it regulates the frequency of Ca^2+^ oscillations that lead to glucose-stimulated insulin secretion by β-cells (Brixel et al., 2010; Ketterer et al., 2011). Moreover, its most iconic role is played in taste-sensing receptor cells, where it is co-expressed with other receptors and signaling molecules involved in the process of bitter, sweet, and umami stimuli detection (Perez et al., 2002; Zhang et al., 2003; Dutta et al., 2018). TRPM5 expression has also been reported in other chemosensory cells in the olfactory, respiratory, and digestive systems, suggesting a broader role in chemosensory processes (Kaske et al., 2007; Bezencon et al., 2008; Lin et al., 2008). The available information regarding TRPM5 trafficking is insufficient to relate this process to specific cellular or tissue failure. However, unlike what has been reported for surface proteins (Khanna et al., 2001), the N-glycosylation would not be involved in the trafficking of TRPM5 to the plasma membrane but rather regulates channel function (Syam et al., 2014).

### TRPM6

TRPM6 is a Ca^2+^ permeable ion channel, which is also highly permeable to Mg^2+^ (Schlingmann et al., 2007). Interestingly, it exhibits an unusual feature, as it contains a transmembrane segment fused to a cytosolic α-type serine/threonine protein kinase domain, which allows it to act as a chanzyme (Chubanov et al., 2018). TRPM6 expression is more restricted than most TRPM channels, with the highest levels of expression reported in the kidney and the gastrointestinal tract, where it is, interestingly, regulated by dietary magnesium (Groenestege et al., 2006; Lameris et al., 2015). The most important role of TRPM6 is related to the homeostasis of systemic Mg^2+^ homeostasis, where different mutations and dysregulation of its activity have been widely reported in patients with different forms of hypomagnesemia (Schlingmann et al., 2002; Chubanov et al., 2007; Lainez et al., 2014) while also being important for the correct functioning of colonic epithelial cells (Luongo et al., 2018).

It has been reported that patients with hypomagnesemia with secondary hypocalcemia present the S141L mutation, which causes a decrease in the expression of TRPM6 at the plasma membrane (Chubanov et al., 2004). Moreover, Bouras *et al.* (2020) showed that short-term exposure to type 2 diabetes mellitus drug metformin (1,1-dimethylbiguanide) increased TRPM6 expression in the plasma membrane while, interestingly, long-term exposure significantly decreased the plasma membrane expression of the channel (Bouras et al., 2020). Another interesting mechanism reported for TRPM6 trafficking suggests activating the EGF receptor increased TRPM6 trafficking to the plasma membrane through the src kinase-Rac1 pathway (Thebault et al., 2009). Moreover, Furukawa et al. suggested that Tumor necrosis factor α (TNFα) could regulate TRPM6 trafficking to the plasma membrane (Furukawa et al., 2017). Additionally, although phosphorylation by the close relative TRPM7 and TRPM6/TRPM7 multimers does not appear to alter TRPM6 trafficking (Brandao et al., 2014), it appears TRPM7 is still necessary for the correct trafficking of TRPM6 to the plasma membrane (Schmitz et al., 2005; Shari et al., 2010).

### TRPM7

TRPM7, although being discovered independently, is very similar in structure and function to its homolog TRPM6, being also a Mg^2+^/Ca^2+^ permeable ion channel (Nadler et al., 2001; Runnels et al., 2001; Schmitz et al., 2003). As with TRPM6, it contains the unique C-terminal serine/threonine protein kinase domain, which shares a low homology with other kinases and is important for the autoregulation of channel activity (Schlingmann et al., 2007; Cabezas-Bratesco et al., 2015; Chubanov et al., 2018). Unlike TRPM6, TRPM7 is widely expressed in many tissues and organs (Nadler et al., 2001; Runnels et al., 2001) but a higher expression has been reported in the heart and kidney (Montell, 2005).

Given its ubiquitous expression, TRPM7 has been involved in several physiological and pathological processes (Visser et al., 2014). For example, in the brain, it regulates synaptic transmission and plasticity, while on the other hand, it also regulates neuronal death following ischemia or neuron injury (Aarts et al., 2003; Krapivinsky et al., 2006; Abumaria et al., 2018). In this context, the fusion of synaptic-like vesicles in PC12 cells is somewhat modulated by vesicular TRPM7 activity (Brauchi et al., 2008). Moreover, it has been reported in several types of cancer, such as breast (Guilbert et al., 2013) and ovarian cancer (Wang et al., 2014), as a metastasis promoter. In contrast, in the heart, it has been involved in forming cardiac fibrosis (Yu et al., 2014).

In the context of TRPM7 trafficking, its most studied regulator is its close homolog, TRPM6. It has been reported that the TRPM6 kinase domain can phosphorylate TRPM7 in serine residues, resulting in altered TRPM7 trafficking. However, TRPM7 phosphorylation of TRPM6 showed no effect on its localization (Schmitz et al., 2005; Brandao et al., 2014).

Interestingly, another regulator of TRPM7 trafficking is TRPM7 itself. It has been shown that S1360 of TRPM7 is a key residue for autophosphorylation, mediating both TRPM7 stability and intracellular trafficking. Other important autophosphorylation residues in the channel were S1403 and S1567, whose autophosphorylation by TRPM7’s kinase activity regulates its interaction with the 14-3-3θ chaperon protein, which regulates channel trafficking (Cai et al., 2018).

TRPM7 channels experience two types of trafficking. The complete gene product traffics to the plasma membrane, where a proteolytic cleavage product containing the kinase domain (M7CKs) is generated and translocated to the nucleus. These fragments establish interactions with proteins composing chromatin remodeling complexes (Krapivinsky et al., 2014). This mechanism is relevant during development (Desai et al., 2012), and the transient overexpression of mouse TRPM7 in HEK293 cells was found to significantly alter the cellular transcription of hundreds of genes (Lee et al., 2011). Finally, it has been shown that sheer stress on blood vessels can increase TRPM7 in the plasma membrane (Oancea et al., 2006).

### TRPM8

TRPM8 is a non-selective Ca^2+^-permeable channel activated by cold, membrane depolarization, and different cooling compounds and is considered the most important thermoreceptor in cold perception (McKemy et al., 2002; Brauchi et al., 2004; Bautista et al., 2007). TRPM8 was initially identified in prostate tissue and reported as upregulated in prostate cancer (Tsavaler et al., 2001). The cold current was first identified in DRG sensory neurons (Reid and Flonta, 2001), and then, the channel was identified, cloned, and described (McKemy et al., 2002).

Although mainly expressed in sensory neurons, channel expression has also been reported in several other tissues and organs, such as the cardiovascular system, lungs, bladder, and urogenital tract (Dhaka et al., 2008; Iftinca and Altier, 2020; Liu et al., 2020). Growing evidence has linked TRPM8 expression and function to several types of cancer, including prostate, lung, and breast cancer (Fuessel et al., 2003; Dhennin-Duthille et al., 2011; Hantute-Ghesquier et al., 2018). Additionally, it has also been involved in the development of neuropathic pain (Proudfoot et al., 2006; De Caro et al., 2019) and irritable bowel syndrome (de Jong et al., 2015).

As one of the most studied TRP channels, there’s a wide variety of articles regarding TRPM8 trafficking and membrane context. It was initially reported that TRPM8 channels are located in lipid rafts (Morenilla-Palao et al., 2009). These overexpressed TRPM8 channels reside in a near membrane compartment, actively recycling in and out of the plasma membrane where its motility is restricted (Veliz et al., 2010; Ghosh et al., 2014). This recycling is associated with the SNARE protein VAMP7 (Ghosh et al., 2016). These recycling modes have been shown to vary in response to channel activation, modulating the number of available channels at the plasma membrane *in vitro* and *in vivo* (Toro and Brauchi, 2015). It is worth mentioning that *in vitro* experiments challenged the later observation (Ghosh et al., 2014). Recently, Cornejo *et al.* (2020) demonstrated that trafficking of TRPM8 along the cold-sensitive peripheral axons depended on the axonal ARF-GEF Golgi-specific brefeldin A-resistance factor 1 (GBF1) (Cornejo et al., 2020).

Moreover, the same group demonstrated that phosphorylation of the channel is also a regulator of TRPM8 trafficking, demonstrating the versatility of mechanisms involved in this process (Rivera et al., 2021). Interestingly, two other factors named TRP channel–associated factors (TCAFs) have been described as interactors of TRPM8 that regulate its trafficking but with opposite effects on TRPM8 gating properties, with TCAF1 decreasing its activity and tumoral properties of prostate cancer cells and TCAF2 increasing it along with also promoting migration of prostate cancer cells (Gkika et al., 2015).

**FIGURE 2:**
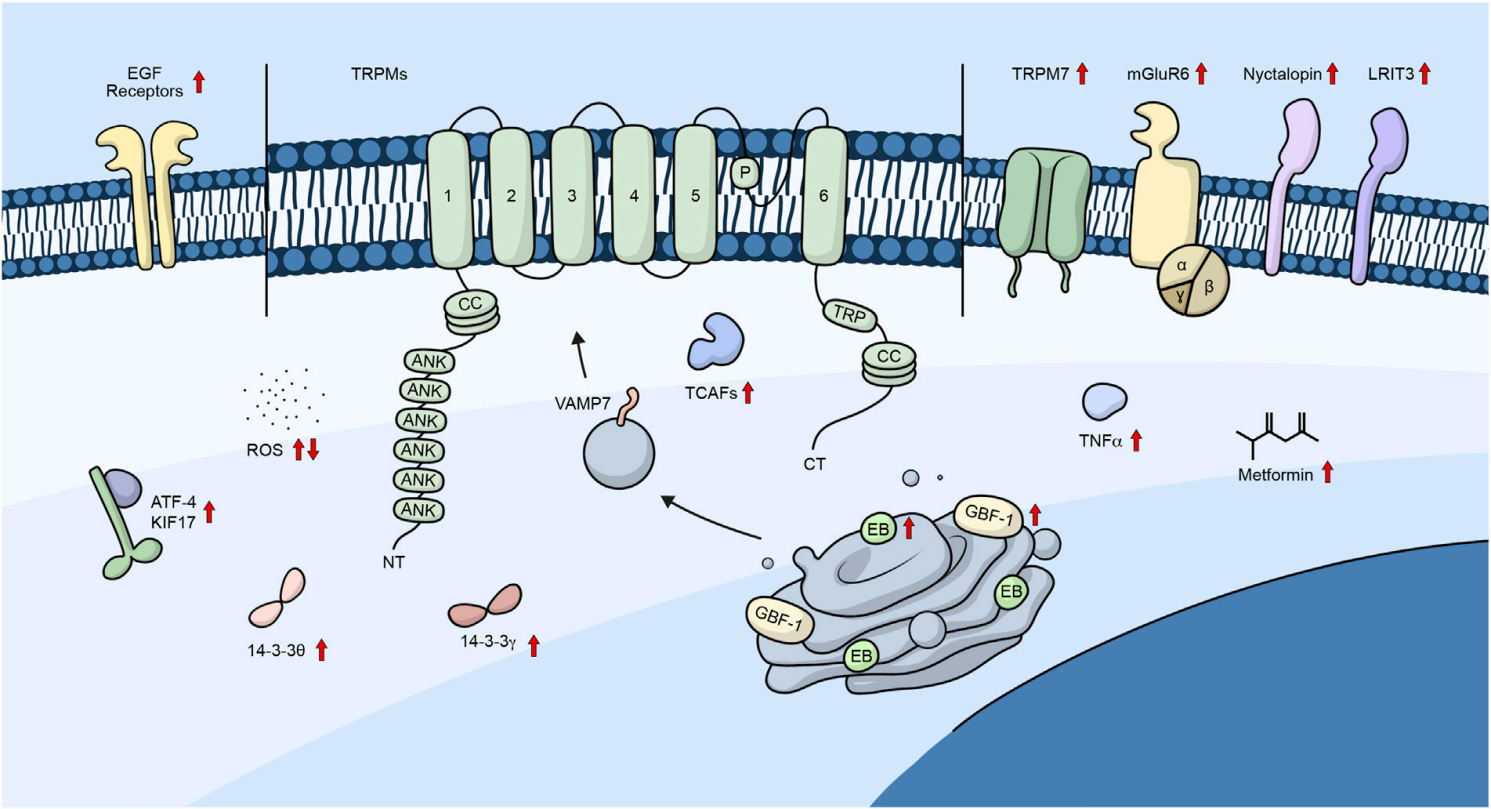
TRPM channels and their trafficking regulators. Diagram showing the different regulators involved in the trafficking of TRPM channels. Embedded in the plasma membrane is a monomeric subunit representing the various domains and regions in TRPM channels. These include ANK (ankyrin repeats), CC (coiled-coil), and TRP, which refers to the TRP box common to all TRP channels. Upward arrows represent regulators that promote channel trafficking to the plasma membrane, whereas downward arrows represent regulators that lower the number of channels in the plasma membrane.

Additionally, Tsuruda *et al.* (2006) demonstrated that the C-terminal of the channel contains a coiled-coil domain that is necessary for channel assembly and trafficking (Tsuruda et al., 2006). On the other hand, it has been suggested that the coiled-coil domain was not necessary for trafficking but rather for channel stabilization and added that the mutation N934Q lowered the glycosylation of the channel and its plasma membrane trafficking. However, it did not prevent it completely, suggesting that glycosylation is not mandatory for channel trafficking (Erler et al., 2006). Moreover, the L1089P mutant prevented channel oligomerization and reduced the surface expression of the channel (Erler et al., 2006). Figure 2 shows a summary of the different proteins/molecules involved in the regulation of the trafficking of TRPM channels to the plasma membrane as were described in this section of the review.

## The TRPV subfamily of ion channels, insights into their trafficking and physiological significance

### TRPV channels and their trafficking

Six subfamilies of TRP channels have been reported in mammals, categorized according to the homology of their sequences. The subfamily of TRPV1-6 (Vanilloid) plays a significant role in all tissues. TRPV1- 4 are thermosensitive and non-selective cation channels, while TRPV5 and TRPV6 are selective to the calcium cation but not sensitive to temperature (Pumroy et al., 2020).

TRPV family has six ankyrin (Ank) repeat domains in the N-terminal and TRP-box in the C-terminal region. The trafficking of TRPV channels is a complex mechanism involving several proteins that vary in the different types of TRPV channels. Studies based on the membrane yeast two-hybrid approach have demonstrated that different trafficking-related proteins interact with TRPV channels, including SNAP-associated protein and synaptotagmin 9, which interact with TRPV1 and TRPV2 (Doñate-Macián et al., 2019). Furthermore, TRPV2 interacts with other proteins, such as lipid phosphatase SAC1, phosphatidylinositide phosphatase, syndecan 3, and SHISHA family member 6, while TRPV4 interacts with Annexin A2 and cyclin-dependent kinase 16 (CDK16) (Doñate-Macián et al., 2019).

Although the trafficking mechanisms of TRPV channels are not yet fully understood, several proteins that interact with these channels play important roles in their trafficking. For example, CDK16 has been found to interact with the coat protein complex II (COPII) complex, allowing the trafficking of TRPV channels (Palmer et al., 2005). Table 3 summarizes some of the known TRPV trafficking-related interactions.

**TABLE 3:** Summary of TRPV trafficking interactions.

Channel	Interaction	Effect	References
TRPV1	SNAP25	delivers to the plasma membrane involving the formation of SNARE complexes	Meng et al. (2016)
	VAMP1	TRPV1/TRPA1 channels are packaged into CGRP- and VAMP1-containing vesicles and delivered to the plasma membrane	Meng et al. (2016)
	Syt IX	TRPV1 co-localizes with Syt IX and the vesicular protein synaptobrevin in vesicles and delivered to the plasma membrane	Morenilla-Palao et al. (2004)
TRPV2	RGA	acts as a chaperone-like protein that controls the translocation of TRPV2 to the plasma membrane in mast cells	Barnhill et al. (2004)
TRPV3	SNX11	promotes the trafficking of TRPV3 from the plasma membrane to lysosomes for degradation via protein-protein interactions	Li et al. (2016)
TRPV4	OS-9	impedes the release of TRPV4 from the ER, resulting in decreased levels of the protein at the plasma membrane	Wang et al. (2007)
	AIP-4	facilitates ubiquitination of TRPV4 and renders the channel available for endocytosis	Wegierski et al. (2006)
	PACSIN 3	regulates endocytosis enhancing the ratio of plasma membrane-associated to cytosolic TRPV4	Cuajungco et al. (2006)
TRPV5	WNK3	complexly glycosylated TRPV5 that appears at the plasma membrane is enhanced by WNK3	Zhang et al. (2008)
	clathrin	TRPV5 internalization	Van de Graaf et al. (2008)
TRPV6	ANNEXIN A2	early endosomes participating in the endocytic pathway of these channels	Gerke and Moss (2002)

## Pathologies associated with trafficking of TRPV channels

### TRPV1

Transient receptor potential vanilloid 1 (TRPV1), the first identified member of the TRPV subfamily, is a tetramer that contains an ATP-binding site and a CaM-binding site in an ankyrin repeat (Sasase et al., 2022). The TRPV1 plays a key role in the perception of peripheral thermal and inflammatory pain.

Yeast two-hybrid screening for identifying proteins that associate with the N terminus of TRPV1 revealed two vesicular proteins, Snapin and synaptotagmin IX (Syt IX). These proteins were found to strongly interact with the TRPV1 N-terminal domain both *in vitro* and *in vivo*, where TRPV1 co-localizes with Syt IX and the vesicular protein synaptobrevin in vesicles. PKC activation leads to a swift delivery of functional TRPV1 channels to the plasma membrane, which suggests that PKC signaling promotes the SNARE-dependent exocytosis of TRPV1 to the cell surface (Morenilla-Palao et al., 2004).

The SNARE family of proteins plays a crucial role in TRPV1 channel trafficking, whose main function is to mediate fusion between membranes (Sudhof and Rothman, 2009). It has been shown that TNFα induced surface trafficking of TRPV1 where Munc18-1, SNAP-25, and VAMP1 are important for transporting TRPV1 to the plasma membrane. During this process, TRPV1 channels are packaged into calcitonin gene-related peptide (CGRP)- and VAMP1-containing vesicles and delivered to the plasma membrane involving the formation of SNARE complexes composed of SNAP-25, syntaxin 1, and VAMP1, as well as Munc18–1 (Meng et al., 2016).

Proinflammatory factors have also been studied for potentiating TRPV1 membrane expression, and it was shown that the SNARE complex mediates TRPV1 exocytosis. These findings suggest that the SNARE complex could be a potential therapeutic target for TRPV1-related disorders (Camprubi-Robles et al., 2009).

Studies have reported that TRPV1 interacts with γ-amino butyric acid A-type (GABAA) receptor-associated protein (GABARAP) in HEK293 cells and neurons. The presence of GABARAP selectively enhances the interaction between tubulin and the C-terminal domain of TRPV1. Moreover, nocodazole treatment reduces the capsaicin-evoked current in cells expressing TRPV1 and GABARAP. This indicates that GABARAP plays an important role in the TRPV1 trafficking and clustering on the plasma membrane (Laínez et al., 2010). Another protein recently described to regulate TRPV1 transport is the cyclin-dependent kinase 5 (CDK5). CDK5 positively controls TRPV1 membrane transport mediating kinesin family member KIF13B-TRPV1 association without altering the total amount of TRPV1. Co-immunoprecipitation experiments confirmed the interaction between TRPV1 and potassium channel auxiliary subunit Kvβ2, exerting a chaperone-like effect that increases the cell surface expression of TRPV1. This increase in TRPV1 cell surface expression is associated with an enhancement in capsaicin sensitivity assay in a heterologous recombinant system in HEK293 cells (Ferrandiz-Huertas et al., 2014b).

TRPV channels have been associated with various pathologies, where many of them are related to the functionality and expression of TRPV channels, including neuropathic pain, migraine, dry eye disease (DED). It is worth mentioning that while TRPV1 functionality may be compromised in these conditions, while its trafficking remains unaffected (Sasase et al., 2022).

### TRPV2

Studies on TRPV channels have revealed the significance of their trafficking to the plasma membrane in various pathologies. Deletion of the distal N-terminus of TRPV2 depletes the channel’s trafficking to the plasma membrane (Donate-Macian et al., 2015).

The recombinase gene activator protein (RGA) has been associated with TRPV2 trafficking. It acts as a chaperone-like protein that controls the translocation of TRPV2 to the plasma membrane in mast cells. RGA is localized to a vesicular sub compartment of the ER/Golgi apparatus and functions as a chaperone-like protein, promoting the membrane trafficking of TRPV2 (Barnhill et al., 2004).

An elevation of cAMP leads to the translocation of TRPV2 to the plasma membrane, indicating that any stimulus that increases this secondary messenger could regulate TRPV2 trafficking (Kanzaki et al., 1999). The rapid exocytotic response induced by insulin is accompanied by membrane insertion of TRPV2 in a PI3K-dependent manner (Aoyagi et al., 2010). Studies on TRPV2 knockout mice have shown decreased systolic function and impaired cardiac functional response to forced treadmill exercise, but the trafficking of these channels in these pathologies remains unclear (Sasase et al., 2022).

### TRPV3

The TRPV3 ion channel is widely expressed in skin keratinocytes, and its exact trafficking mechanism to/from the plasma membrane is unknown. It has been reported that the vesicular trafficking protein sorting nexin 11 (SNX11) downregulates the TRPV3 plasma membrane protein level. SNX11 interacts with TRPV3 and promotes the trafficking of TRPV3 from the plasma membrane to lysosomes for degradation *via* protein-protein interactions (Li et al., 2016).

TRPV3 plays an important regulatory role in temperature perception, pain transduction, skin physiology, inflammation, cancer, and other diseases, such as pruritic and atopic dermatitis, psoriasis, rosacea, myocardial hypertrophy where the overexpression of this channel is involved (Su et al., 2023). However, there is currently no evidence suggesting that TRPV3 trafficking is altered in these conditions.

### TRPV4

The interaction between the N-terminus of TRPV4 and the ubiquitously expressed ER-associated protein OS-9 prevents the channel from trafficking to the plasma membrane. This interaction impedes the release of TRPV4 from the ER, resulting in decreased protein levels at the plasma membrane. Furthermore, OS-9 appears to bind preferentially to immature variants and monomers of TRPV4 are located in the ER and attenuate their polyubiquitination (Wang et al., 2007). Moreover, it has also been reported that deleting the C-terminal region causes retention in the ER (Becker et al., 2008). Taken together, these findings suggest that the process of TRPV channel trafficking is a complicated process that involves several proteins, which may vary depending on the specific type of TRPV channel involved. In the context of TRPV channel mutations, TRPV4 serves as an example of a channel associated with various channelopathies resulting from mutations in different channel motifs, including both the C and N terminal regions and the pore. Some of these mutations have been shown to affect channel conductivity, while others may interfere with proper channel trafficking due to their location in the N- and C-terminal regions, which are essential for this process (Verma et al., 2010). For instance, a mutation of Asn-651 into Gln has been found to increase the constitutive membrane trafficking of TRPV4 without affecting overall channel expression. This mutation is situated in the consensus N-linked glycosylation motif between S5 and S6, suggesting that glycosylation of this residue could regulate channel trafficking (Xu et al., 2006). Additionally, phosphorylation of two other residues, S824 (Shin et al., 2012) and Y110 (Wegierski et al., 2009), is important for their abundance in the plasma membrane and proper trafficking of TRPV4. The atrophin-interacting protein 4 (AIP-4) facilitates ubiquitination of TRPV4 and renders the channel available for endocytosis (Wegierski et al., 2006). The interaction of TRPV4 with PACSIN 3 isoform regulates endocytosis enhancing the ratio of plasma membrane associated to cytosolic TRPV4 (Cuajungco et al., 2006). In HEK293 and vascular endothelial cells, depletion of intracellular Ca^2+^ stores leads to the vesicular trafficking of heteromeric TRPV4-C1 channels to the plasma membrane (Ma et al., 2010).

In contrast, it has been observed that splicing variants of TRPV4, which lack the ankyrin domain, do not oligomerize and are retained in the ER (Arniges et al., 2006). Some of these variants have been detected in two unrelated human respiratory tracts (CFT1-LCFSN and HBE epithelial cell lines), highlighting the significance of these channels in the respiratory tract and, therefore, potentially associated pathologies.

### TRPV5

Deleting the distal TRPV5 N-terminus is sufficient to deplete the channel trafficking to the plasma membrane (Donate-Macian et al., 2015). Moreover, the complexly glycosylated TRPV5 that appears at the plasma membrane is enhanced by WNK3, a member of the With No Lysine (K) family of protein serine/threonine kinases. The microtubule inhibitor colchicine blocks the effect of WNK3 on TRPV5, indicating that WNK3 positively regulates the transcellular Ca^2+^ transport pathway by increasing the exocytosis of TRPV5 (Zhang et al., 2008). Conversely, TRPV5 internalization occurs through the clathrin protein (van de Graaf et al., 2008).

Familial hypomagnesemia with hypercalciuria and nephrocalcinosis (FHHNC) is a paracellular channelopathy caused by mutations in the claudin-16 and claudin-19 genes. The missense FHHNC mutation c.908C>G (p.T303R) in the claudin-16 gene disrupts the phosphorylation of the claudin-16 protein. The phosphomimetic claudin-16 protein carrying the T303E mutation, but not the wildtype claudin-16 or the T303R mutant protein, increases the TRPV5 channel conductance and membrane abundance in human kidney cells (Hou et al., 2019).

### TRPV6

Annexin 2 also interacts with TRPV6 (Borthwick et al., 2008). Its known functions include intracellular trafficking of vesicles, maintenance and organization of cell membranes. Annexin A2 was also identified on early endosomes participating in the endocytic pathway of these channels (Gerke and Moss, 2002).

Pharmacological modulation of TRPV channels can treat certain pathologies by affecting their functionality (Seebohm and Schreiber, 2021). Nevertheless, mutations in these channels can also affect their trafficking, leading to various diseases, which will be described below.

Mutations in the TRPV6 channel have been studied and found to cause insufficient Ca^2+^ transport, resulting in transient neonatal hyperparathyroidism (TNHP) characterized by respiratory distress and under-mineralization of the skeleton. Two mutations, I223T and G428R, were identified in TRPV6 that affect its trafficking to the plasma membrane. The I223T mutation is located in the fourth ankyrin repeat domain (ANK4) and decreases the amount of TRPV6 channel in the plasma membrane (Yamashita et al., 2019).

Similarly, the G428R mutation located in the S2 and S3 transmembrane domains also affects trafficking to the plasma membrane and interferes with TRPV6’s normal function. We thus conclude that interference with placental maternal-fetal calcium transport caused by TRPV6 loss-of-function mutations results in fetal calcium deficiency, hyperparathyroidism, and metabolic bone disease (Suzuki et al., 2018).

**FIGURE 3:**
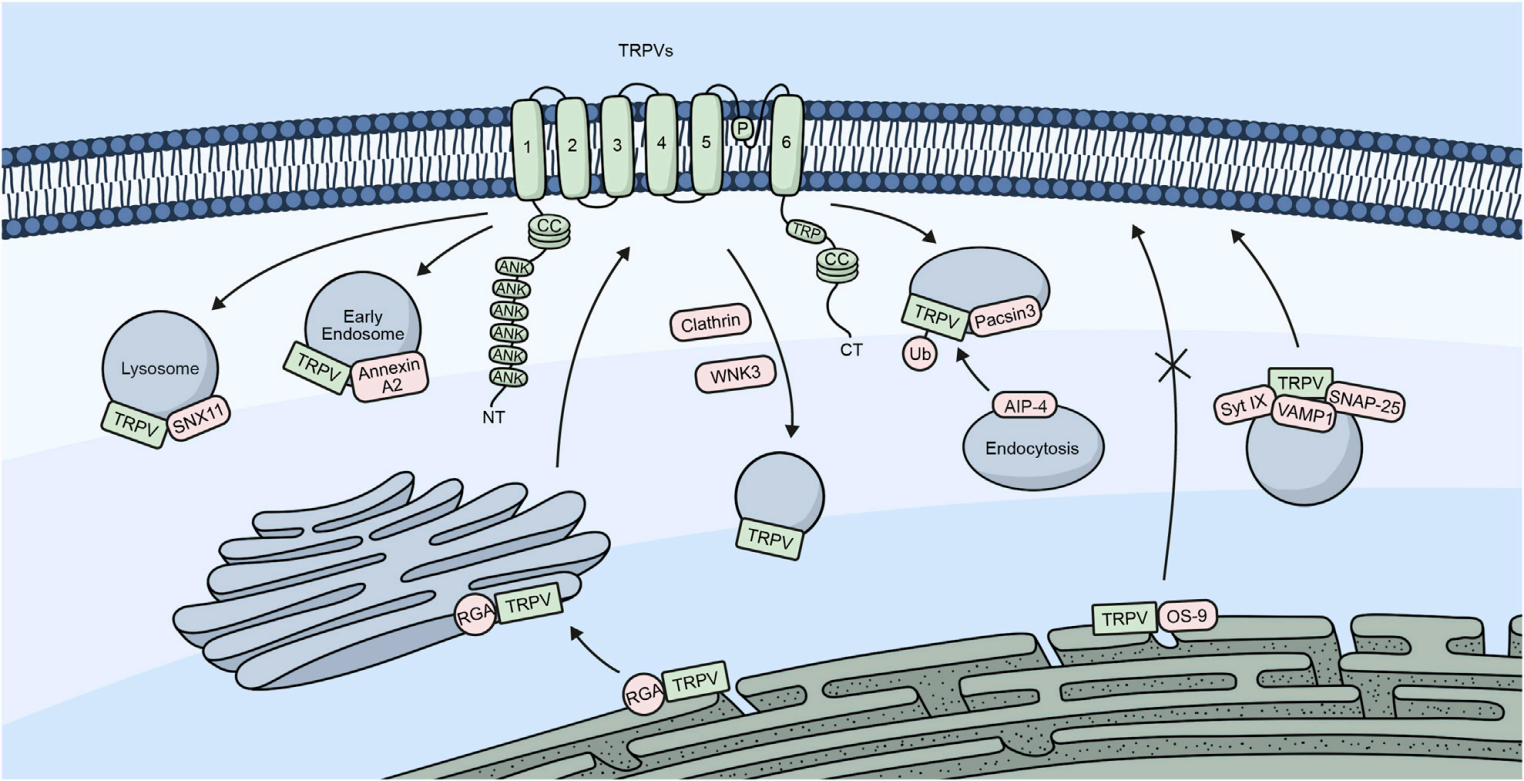
TRPV channels and their trafficking regulators. Embedded in the plasma membrane is a monomeric subunit representing the different domains and regions in TRPV channels. These include ANK (ankyrin repeats), CC (coiled-coil), and TRP, which refers to the TRP box common to all TRP channels. The diagram shows a summary of the different regulators involved in the trafficking of TRPV channels.

Figure 3 shows a summary of the different proteins/molecules involved in the regulation of the trafficking of TRPV channels to the plasma membrane.

## TRPA1 in nociception: function, membrane expression, and sensitization mechanisms

TRPA1 was initially identified as a nociceptive channel that is expressed in subpopulations of primary sensory neurons in the DRG, vagal ganglia (VG), and trigeminal ganglia (TG), which is activated by noxious compounds and low temperatures (Story et al., 2003; Bautista et al., 2005). TRPA1 is predominantly expressed in unmyelinated C-fibers and thinly myelinated Aδ-fibers, which transmit pain signals from the periphery to the central nervous system. TRPA1 expression in large, myelinated fibers is less frequent, and its function in these fibers is poorly understood (Story et al., 2003). This selective expression pattern suggests that TRPA1 plays a specific role in nociception, which is the perception of pain and helps to ensure that noxious stimuli are transmitted effectively to the central nervous system.

The expression of TRPA1 can vary between species, with some species having a higher expression of TRPA1 in certain populations of primary sensory neurons. At least 25% of TRPA1-expressing neurons are peptidergic, which means they can release substance P (SP) and CGRP. However, TRPA1 has also been found to colocalize with non-peptidergic neuron markers, such as isolectin B4 (IB4), the purinergic P2X3 receptor, and the Na_V_1.8 channel, which suggests that TRPA1 plays a complex role in nociception and other physiological processes (Kim et al., 2010; Kim et al., 2011; Ryskamp et al., 2011).

TRPA1 is present in 30%–50% of TRPV1-expressing neurons (Kobayashi et al., 2005). Evidence for a TRPV1-TRPA1 interaction suggests that the two proteins might form a heteromeric channel, which could play a role in nociception and other physiological processes (Fischer et al., 2014). These two channels are thought to play a crucial role in transmitting sensory signals during inflammation, as they were found on vesicles containing CGRP and co-localized on nerve fibers and sensory neuron cell bodies. Furthermore, TNFα was observed to increase the surface presence of TRPV1 and TRPA1, indicating upregulation or enhanced transport to the cell membrane (Meng et al., 2016; Nugent et al., 2018). The proximity of TRPV1 and TRPA1 suggests potential co-trafficking in response to TNFα. Notably, Munc18–1 and VAMP1 were identified as essential for TNFα-induced channel trafficking and CGRP release. Additionally, Botulinum neurotoxins, particularly BoNT/C1 or/A, effectively inhibited TNFα-induced channel delivery, indicating their therapeutic potential in mitigating sensitization during inflammation (Wu C. et al., 2016; Meng et al., 2016). The trafficking of TRPA1 is regulated by interactions with several proteins, as summarized in Table 4.

**TABLE 4:** Summary of TRPA trafficking interactions.

Channel	Interaction	Effect	References
TRPA1	TNFα	increases the surface presence of TRPA1/TRPV1 complex	Meng et al. (2016), Nugent et al. (2018)
	BONT/C1 and A (Botulinum neurotoxin C1 and A)	inhibits TNFα-induced channel trafficking to the membrane	Meng et al. (2016), Wu et al. (2016b)
	SNAP25	delivers to the plasma membrane involving the formation of SNARE complexes	Meng et al. (2016)
	VAMP1	TRPV1/TRPA1 channels are packaged into CGRP- and VAMP1-containing vesicles and delivered to the plasma membrane	Meng et al. (2016)
	Munc18–1	TNFα induces co-trafficking of TRPV1/TRPA1 in VAMP1-containing vesicles to the plasmalemma via Munc18–1/syntaxin1/SNAP-25 mediated fusion	Meng et al. (2016)
	IQGAP1	promotes chronic pain by interaction with TRPA1, increases trafficking to the neuronal membrane, catalyzed by the small GTPase Cdc42	Khan et al. (2023)
	GTPase Cdc42	catalyzes interaction of IQGAP1 with TRPA1, promoting the membrane trafficking	Khan et al. (2023)
	AKAP (A-Kinase Anchoring Protein) 79/150	anchoring of PKA/PKC to TRPA1 in the plasma membrane organizes post-translational receptor modifications	Brackley et al. (2017)
	Sigma-1 receptor	interacts with TRPA1, leading to chemotherapy-induced painful peripheral neuropathy (CIPN)	Marcotti et al. (2022)
	Sigma-1 receptor antagonist (S1RA)	inhibits the plasma membrane expression and function of human TRPA1 by disrupting the interaction between Sigma-1R and TRPA1	Marcotti et al. (2022)
	cyclin-dependent kinase 5 (Cdk5)	phosphorylates various sites of TRPA1, leading to trafficking to the plasma membrane	Hall et al. (2018), Hynkova et al. (2016), Sulak et al. (2018)

Inflammation can sensitize peripheral nociceptors and cause symptoms such as allodynia and hyperalgesia. Bradykinin is one of the key inflammatory mediators known to sensitize TRPA1 ion channels. The sensitization of TRPA1 is dependent on PLC and PKA activation, but not PKC activation. It diminishes the nociceptor activation thresholds, which can result in symptoms of allodynia and hyperalgesia (Wang et al., 2008).

The effect of PLC on sensitization may involve hydrolysis of PIP_2_ (Dai et al., 2007; Karashima et al., 2008; Kim et al., 2008). Both PKA and PLC activation increase the membrane expression of TRPA1 in trigeminal neurons by increasing the trafficking of the channel (Schmidt et al., 2009). PKA-mediated sensitization of TRPA1 has been confirmed in some studies, with three residues in the NH2-terminal domain (S86, S317, and S428) and one residue in the COOH-terminal domain (S972) identified as the main phosphorylation sites for PKA (Meents et al., 2017). Other studies have also investigated the phosphorylation of TRPA1 by cyclin-dependent kinase 5 (Cdk5), with sites S242, T416, S449, T485, and T673 identified as susceptible phosphorylation sites (Hynkova et al., 2016; Hall et al., 2018; Sulak et al., 2018). During TRPA1 phosphorylation by both PKA and PKC, the anchoring protein AKAP (A-Kinase Anchoring Protein 79/150) is essential in anchoring these kinases to TRPA1 (Brackley et al., 2017). TRPA1 contains an N-terminal ankyrin repeat domain (ARD) with a total of 17 ankyrin repeats. This domain is believed to be essential for plasma membrane localization (Nilius et al., 2011; Hall et al., 2018). TRPA1 phosphorylation may contribute to painful pathologies associated with the channel, including chemotherapy-induced peripheral neuropathy (CIPN).

Sigma-1 receptor (Sigma-1R), an endoplasmic reticulum chaperone, is known to modulate the function of various ion channels and receptors (Hanner et al., 1996; Alonso et al., 2000; Delprat et al., 2020). Sigma-1 receptor antagonist (S1RA) has been shown to interact with TRPA1. Antagonizing Sigma-1R disrupted the formation of the molecular complex between Sigma-1R and TRPA1, affecting the trafficking of TRPA1 to the plasma membrane. Moreover, S1RA significantly inhibited the plasma membrane expression and function of human TRPA1 channels, reducing the excitability of nociceptor neurons sensitized by oxaliplatin (Marcotti et al., 2023).

TRPA1 channels were also shown to sense moderate hypoxia in astrocytes associated with the anterior inferior cerebellar artery (AICA) in the parafacial respiratory group/retrotrapezoid nucleus (pFRG/RTN) region of the ventral medullary surface. TRPA1 prevents its internalization through prolyl hydroxylases (PHD) and neural precursor cell-expressed developmentally downregulated protein 4 (NEDD4-1)-mediated mechanisms, resulting in plasma membrane retention. Hypoxia-induced TRPA1 channel activity leads to Ca^2+^ influx. It accelerates ATP release from pFRG/RTN astrocytes, impacting neuronal circuits in the respiratory center and increasing the amplitude of the inspiratory discharge rhythm. This study suggests a connection between TRPA1 trafficking regulation and the cellular response to hypoxia, highlighting the role of TRPA1 in astrocytic sensing of acute hypoxia (Uchiyama et al., 2020).

Recently, IQ motif containing GTPase activating protein 1 (IQGAP1) was shown to interact with TRPA1, mainly in sensory dorsal root ganglia neurons in mice. The expression of IQGAP1 increases in chronic pain conditions, and TRPA1 undergoes increased trafficking to the neuronal membrane, catalyzed by the small GTPase Cdc42, which is associated with IQGAP1. Activation of PKA can also induce TRPA1 trafficking and sensitization. Notably, the absence of IQGAP1 prevents the responses, implying a mechanism entirely dependent on the presence of IQGAP1. These findings suggest the possibility of IQGAP1 in promoting chronic pain by facilitating the trafficking and signaling mechanisms of TRPA1 channels (Khan et al., 2023). Altogether, TRPA1 channels are crucial in pain perception and potentially affect chronic pain conditions.

## Concluding remarks

The TRP family of ion channels plays an essential role in many physiological processes by regulating intracellular levels of various ions, such as sodium, calcium, and magnesium. Indeed, the dysregulation of the activity of these channels can contribute to and/or lead to the development of several pathological disorders, such as cancer, brain diseases, and heart and kidney malfunction. Interestingly, one of the primary means that cells must regulate the activity of TRP channels is the modulation of the number of channels in the plasma membrane. In this review, we have presented an in-depth exploration of the mechanisms that have been described for the trafficking of the different members of the TRP family of ion channels and the pathologies associated with the dysregulation of these processes. In this context, although these channels share a high homology in their amino acid sequence, we have found that their trafficking mechanisms are widely different regarding interacting proteins and signaling pathways involved, even amongst channels of the same sub-family, which provides the intracellular targeting characteristic of these channels.

Nevertheless, we can point to specific general mechanisms shared amongst TRP channels necessary for correct trafficking and localization. For example, post-translational modifications such as glycosylation, phosphorylation, and SUMOylation are involved in the trafficking of several TRP channels. On the other hand, protein-protein interactions seem to be of great importance for the trafficking of TRP channels, where proteins such as PKA, PKC, and PLC are common regulators of the localization of some members of this family. Additionally, the subcellular microenvironment also appears to be an important feature for the trafficking of several TRP channels.

As a final remark, we have also briefly described the wide variety of processes and pathologies in which TRP channels are highly involved. In recent years, several reports have appeared showing the therapeutical potential that targeting these ion channels through modulators and other mechanisms might have (Moran et al., 2011; Zsombok and Derbenev, 2016; Hong et al., 2020; Fallah et al., 2022; Koivisto et al., 2022). In general, most of these studies have been dedicated to the identification of novel pharmacological modulators addressed to the conductive activity of these channels. However, ion channels can also have non-conductive activities, serving as scaffolding proteins and signaling hubs in different processes. Then, their specific trafficking and localization might regulate these functions. Therefore, further studies dedicated to targeting the trafficking and targeting of TRP channels might serve as potential therapeutic strategies for different diseases.

In conclusion, the trafficking of TRP channels is characterized by its intricate nature, the different mechanisms involved, such as vesicular transport, anterograde, and retrograde pathways, and the many diseases associated with this process’s dysregulation. The present overview underscores the importance of expanding our understanding of the mechanisms involved in the trafficking of TRP channels to provide novel therapeutic targets and treatments for the increasing number of pathologies associated with the TRP family of ion channels.
